# The Effects of Mind–Body Exercise on the Management of Fibromyalgia Symptoms: A Systematic Review and Meta‐Analysis

**DOI:** 10.1155/prm/9436329

**Published:** 2026-11-15

**Authors:** Ping-an Zhu, Nan Kan, Juan Guo, Howe Liu, Yuan-Yuan Sun, Yue-wen Ma

**Affiliations:** ^1^ Department of Rehabilitation Medicine The First Affiliated Hospital of China Medical University Shenyang China cmu.edu.cn; ^2^ Department of Physical Therapy Louisiana State University Health Science Center School of Allied Health Professions New Orleans Louisiana USA; ^3^ Department of Medical Genetics China Medical University Shenyang China cmu.edu.tw

**Keywords:** fibromyalgia, meta-analysis, mind–body exercise

## Abstract

**Background:**

The FM guidelines recommend that the treatment of FM should be based on nondrug treatment. Currently, more and more forms of MBE are being applied to the management of FM symptoms. However, the efficacy of the different MBE forms is not known.

**Methods:**

Five electronic databases were searched in the period ending Jan 20, 2026. Randomized controlled trials were selected and screened according to inclusion and exclusion criteria.

**Results:**

A total of 20 studies involving 1177 patients with FM were included. MBE significantly improved core symptoms in patients with FM compared to control conditions.

**Subgroup Analysis:**

For pain reduction, both TC/qigong and Pilates/yoga showed significant benefits (all *p* < 0.01), with no statistically significant difference between the two subgroups (*p* = 0.23). Regarding the overall impact of FM, both TC/qigong and Pilates/yoga significantly reduced disease impact (all *p* < 0.01). The difference between subgroups approached but did not reach statistical significance (*p* = 0.05). For anxiety symptoms, both MBE subtypes were associated with significant improvements (all *p* < 0.05), with no significant difference observed between TC/qigong and Pilates/yoga. In terms of depressive symptoms, TC/qigong demonstrated significant improvement (*p* < 0.01), whereas Pilates/yoga did not yield a significant effect (*p* = 0.17). However, the difference between subgroups was not statistically significant (*p* = 0.14). Significant improvements were also observed for sleep quality (*p* = 0.01), fatigue (*p* = 0.001), and quality of life (*p* < 0.01) following MBE interventions.

**Conclusion:**

MBE significantly improves pain, fatigue, sleep, anxiety, depression, and quality of life in fibromyalgia patients. Subgroup analyses indicate that TC/qigong and Pilates/yoga show comparable efficacy in pain and anxiety reduction, while TC/qigong may offer additional benefits for depressive symptoms. These findings provide updated evidence with broader outcome coverage and larger average sample sizes compared to previous meta‐analyses.

## 1. Introduction

Fibromyalgia (FM) is a chronic syndrome. Its main symptoms are chronic and extensive musculoskeletal pain, fatigue, sleep disorders, and prominent physiological and psychological impairments [1]. These symptoms eventually can seriously affect the patient’s quality of life [2]. The prevalence rate of FM in the general population is 0.2%–6.6%, which is mostly seen in women [3]. Many studies estimate that the prevalence of FM is as high as 4.9% in women and 2.9% in men [4–6]. At present, studies have shown that FM causes direct costs (such as drug treatment and other medical care expenses) and indirect costs (such as loss of productivity) to increase [7]. The pathogenesis of FM is controversial. Some studies believe that it might be a multifactorial result from genetic, environmental, psychological, neuropathic, and/or nutritional regulation factors [8], among which the most credible mechanism might be pain regulation and central sensitivity disorder [1, 9, 10].

Nowadays, FM is not able to be cured. The FM guidelines issued by the European Rheumatology Union in 2017 [11] and the Canadian Pain Association and the Canadian Rheumatology Association in 2013 [12] suggest that the treatment for FM should focus on nondrug treatment, and the available interventions should be tailored according to the intensity of symptoms and the preferences of patients. Therefore, it is urgent to determine effective nondrug interventions for FM patients. In the past few decades, complementary and alternative medicine (CAM) has become very popular among patients with various diseases. Mind–body exercise (MBE) has been included in CAM. With the development of research technology, more MBE forms are applied to the management of FM symptoms. tai chi (TC) [13], Pilates [14], yoga [15], and qigong [16] are currently the four most popular MBE modes.

TC and qigong are both ancient Chinese traditional martial arts. As an MBE model, TC is a physical and mental exercise that uses physical movement and breathing control to improve breathing ability, balance, and posture control and reduce the pressure and anxiety of the elderly [17–19]. Qigong allows participants to learn how to regulate, integrate, and cultivate the body, breathing (such as deep breathing involving the whole body), and mind (e.g., mental relaxation) [20]. Pilates is a kind of exercise method based on the teachings of Joseph Pilates (1880–1967). Its main characteristics are concentration, control, accuracy, breathing and flow, aiming at improving the overall flexibility of the body, core strength, posture, and coordination of breathing and movement [21]. Yoga is an ancient oriental practice that combines body posture, breath control, and meditation [22]. These four MBE models have been used to treat different chronic pain conditions [23–26], but the results of these studies are mixed. Previous studies have shown that TC can reduce the symptoms of FM patients compared with the control group, for example, including pain, sleep, depression, anxiety, and quality of life [25]. Similarly, a randomized controlled trial (RCT) study found that Pilates can effectively improve the pain and anxiety of FM patients [27]. However, there are other MBE models that have not reached similar results [28, 29]. Recent studies have investigated the overall efficacy of MBE for FM [30]. However, these studies have primarily compared various mind–body interventions against control groups, without directly contrasting Chinese medicine–based practices (TC and qigong) with Western rehabilitation–oriented regimens (Pilates and yoga) in head‐to‐head subgroup analyses. Moreover, while previous meta‐analyses used mean difference (MD) as the effect size (ES), thereby restricting inclusion to studies employing identical assessment tools, we adopted standardized mean difference (SMD) to synthesize evidence from trials using diverse instruments, broadening the evidence base. Our study also systematically evaluated anxiety and fatigue, outcomes inadequately covered in prior reviews. In terms of evidence volume, our analysis includes RCTs with a larger average sample size than earlier meta‐analyses, ensuring greater statistical power. Thus, the incremental contributions of this work are (1) directly comparing, via subgroup analysis, the differential efficacy of TC/qigong versus Pilates/yoga in FM patients; (2) incorporating a wider range of evidence through SMD pooling; (3) systematically assessing anxiety and fatigue; and (4) including English‐language RCTs published up to January 2026, yielding a pooled evidence base with larger average sample sizes. The aforementioned differences highlight the unique positioning and incremental contribution of this study. Therefore, this study aims to conduct a systematic review and meta‐analysis of existing RCT evidence integrating MBE to determine the effectiveness of four of the most popular MBE modalities—TC, qigong, Pilates, and yoga—for patients with FM, focusing on pain, fatigue, depression, anxiety, sleep, and quality of life.

## 2. Methods

### 2.1. Protocol and Registration

This study conformed to the Preferred Reporting Items for Systematic Reviews and Meta‐Analyses (PRISMA) guidelines [31]. The study is registered with the PROSPERO database under registration number CRD4202340209.

### 2.2. Search Strategy

Literature was retrieved through 5 electronic databases (PubMed, Embase, Cochrane, PEDro, and Web of Science). The time cutoff was January 20, 2026, with no specific restrictions on language and country. The following keywords were used: FM, TC, Tai Ji, yoga, qigong, and Pilates. The complete search string can be found in Supporting Table S1–3.

### 2.3. Inclusion and Exclusion Criteria

According to PRISMA 2020 requirements [31], eligibility for the study should include (1) Subjects: Participants were aged 18–80 years and were diagnosed with FM by physicians according to the American College of Rheumatology (ACR) 2010 criteria [13] or the ACR 1990 criteria. (2) Types of intervention: Interventions were the prescription of MBE training, including TC, yoga, Pilates and/or qigong. (3) Outcomes: Primary outcome: Measure the pain intensity after intervention. Secondary outcomes: indicators to evaluate function and overall impact, fatigue, depression, anxiety, sleep, or quality of life after intervention. (4) Types of control group: A control group of any form (e.g., waitlist, physical exercise, and education) other than MBE. (5) Design of studies: RCT. The following conditions were excluded (1) unable to get full text or data; (2) non‐RCTs; and (3) articles published in the form of conference summaries and research programs.

### 2.4. Data Extraction

The information characteristics of each included study were first extracted by two experienced researchers (ZPA and KN). The information extracted by the two researchers includes publication information (e.g., author, year, and country), participant characteristics (e.g., age and sample size), grouping characteristics (e.g., MBE model, intervention time, and follow‐up time), and study type and outcome indicators.

We used change scores (postintervention minus baseline) for the meta‐analysis. When change scores were directly reported with means and standard deviations (SDs), we extracted them directly. When only baseline and postintervention values were reported, we calculated the SD of the change score using the correlation coefficient method recommended by the Cochrane Handbook (assuming a correlation coefficient of *r* = 0.5). Baseline values were used solely for describing participant characteristics (Table 1) and were not directly included in the meta‐analysis. If an article presented data only in graphical form, we used Engauge Digitizer 10.8 software to extract the numerical values; when standard errors (SEs), confidence intervals (CIs), or interquartile ranges (IQRs) were reported instead of SDs, we used the RevMan 5.3 calculator to convert them to SDs. The characteristic forms completed independently by the two researchers were then submitted to the third researcher (MYW) for review, and the final version was produced after consensus.

**TABLE 1:** Characteristics of the included studies.

Intervention	Author	Country	Study design	Sample size (N)	Age (years) (M ± SD)	Outcome	Time of assessment
Pilates.	Ekici 2016	Turkey	RCT	Experimental group: 15 Control group: 21	Experimental group: 37.13 ± 6.37 Control group: 36.86 ± 7.73	VAS FIQ STAI	0/4 weeks
	Medeiros 2020	Brazil	RCT	Experimental group: 21 Control group: 21	Experimental group: 50.7 ± 9.7 Control group: 50.7 ± 9.7	VAS FIQ SF–36 PSQI	0/12 weeks
	Altan 2009	Turkey	RCT	Experimental group: 25 Control group: 24	Experimental group: 48.20 ± 6.5 Control group: 48.20 ± 6.5	VAS FIQ	0/12/24 weeks (follow‐up)
	Sari 2025	Turkey	RCT	Experimental group: 15 Control group: 15	Experimental group: 43.6 ± 6.23 Control group: 47.27 ± 6.4	FIQ FESS NRS–PI HADS	0/8 weeks

Yoga	Schmidt 2011	Germany	RCT	Yoga group: 53 Control group: 59	Yoga group: 53.4 ± 8.7 Control group: 52.3 ± 10.9	FIQ CES‐D STAI PSQI PPS PLC	0/8/16 weeks (follow‐up)
	Carson 2010	United States	RCT	Yoga group: 25 Control group: 28	Yoga group: 51.4 ± 13.7 Control group: 55.8 ± 8.9	FIQR	0/8 weeks
	Şevgin 2025	Turkey	RCT	Aerobic exercise and yoga: 20 aerobic exercise: 20	Aerobic exercise and yoga: 25.4 ± 1.7 aerobic exercise: 24 ± 2.9	FIQ SMPQ	0/12 weeks

Qigong	Sarmento 2020	United States	RCT	Qigong group: 10 Control group: 10	Qigong group: 42.6 ± 10.7 Control group: 56.1 ± 12.3	VAS FIQR PSQI QOLS HADS	0/10 weeks
	Lynch 2012	Canada	RCT	Qigong group: 53 Control group: 47	Qigong group: 52.81 ± 8.91 Control group: 52.13 ± 8.56	NRS–PI FIQ PSQI SF–36	0/8/16 weeks
	Liu 2012	United States	RCT	Qigong group: 6 Control group: 6	Qigong group: 55.7 Control group: 57.5	SMPQ MFI–20 PSQI FIQ	0/6 weeks
	Haak 2008	Sweden	RCT	Qigong group: 29 Control group: 28	Qigong group: 54.0 ± 9.4 Control group: 53.4 ± 8.0	STAI BDI WHOQOL–BREF VNS	0/7 weeks
	Heiresh 2025	Iran	RCT	Qigong group: 17 Control group: 17	Qigong group:65.65 ± 0.70 Control group:65.71 ± 3.05	FIQR PSQI	0/12 weeks
	Juan 2025	Spain	RCT	Qigong group: 18 Control group: 24	Qigong group:65.65 ± 0.70 Control group:65.71 ± 3.05	FIQ DASS–21 VAS SWLS	0/4 weeks
	Astin 2003	United States	RCT	Qigong group: 32 Control group: 33	—	SF–36 FIQ BDI	0/8/14/24 Weeks

Tai chi	Hammond 2006	United Kingdom	RCT	Tai chi group: 71 Control group: 62	Tai chi group: 48.36 ± 10.91 Control group: 48.73 ± 10.95	FIQ	0/12 weeks
	Wang 2010	United States	RCT	Tai chi Group: 33 Control group: 33	Tai chi Group: 49.7 ± 11.8 Control group: 50.5 ± 10.5	FIQ VAS PSQI CES‐D SF–36	0/16/32 weeks
	Wang 2018	United States	RCT	Tai chi group: 36 Control group: 75	Tai chi group: 52.1 ± 13.3 Control group: 50.9 ± 12.5	FIQR VAS HADS PSQI SF–36	0/12/24 weeks
	Wong 2017	United States	RCT	Tai chi group: 17 Control group: 14	Tai chi group: 51 ± 2 Control group: 51 ± 2	VAS	0/12 weeks
	Bongi 2016	Italy	RCT	Tai chi group: 22 Control group: 22	Tai chi group: 51.4 ± 6.8 Control group: 51.4 ± 6.8	FIQ SF–36 FACIT–F WPI PSQI HADS	0/16 weeks
	Jones 2012	United States	RCT	Tai chi group: 51 Control group: 49	Tai chi group: 17.0 Control group: 19.8	FIQ BPI PSQI	0/12 weeks

*Note:* PLC: Quality‐of‐Life Profile for the Chronically Ill; QOLS: Quality‐of‐Life Scale; SMPQ: Short‐Form McGill Pain Questionnaire; MFI–20: Multidimensional Fatigue Inventory; WHOQOL–BREF: World Health Organization Quality of Life–Brief; FACIT–F: Functional Assessment of Chronic Illness–Fatigue; DASS–21: The questionnaires used were the Depression Anxiety Stress Scales; SWLS: the Life Satisfaction Scale. FIQR: Revised Fibromyalgia Impact Questionnaire; STAI: State Anxiety Inventory.

Abbreviations: BDI, Beck’s Depression Inventory; BPI, Brief Pain Inventory; CES‐D, Center for Epidemiologic Studies Depression; FIQ, Fibromyalgia Impact Questionnaire; HADS, Hospital Anxiety and Depression Scale; NRS–PI, Numerical Rating Scale–pain intensity; PPS, Pain Perception Scale; PSQI, Pittsburgh Sleep Quality Index; RCT, randomized controlled trial; SF–36, Short Form–36; VAS, Visual Analog Scales; VNS, Visual Numerological Scale; WPI, Widespread Pain Index.

### 2.5. Quality Assessment

The two authors (ZPA and GJ) independently evaluated the quality of the method and the risk of bias included in the study through the Cochrane Bias Risk Tool and checked seven items, including selection bias, performance bias, detection bias, attrition bias, reporting bias, and other biases. The tool divides quality risk into three categories: low, high, and unclear.

### 2.6. Data Synthesis and Analysis

RevMan5.4.1 statistical software is used for data analysis. The ES was calculated by the SMD of the clinical scale change score and its 95% CI. The 95% CI was evaluated by the *z*‐test. *p* value and *I* ^2^ test were used for the heterogeneity between groups. If *p* < 0.05 and *I* ^2^ > 50%, it indicates that there is heterogeneity between studies. To ensure the reliability of the results, sensitivity analysis is conducted. The random effect model was used for analysis. In addition, for subgroup analysis, the four MBE modalities were categorized into two groups based on their intrinsic attributes: TC/qigong and Pilates/yoga. The rationale for this classification is as follows. Firstly, both TC and qigong originated from traditional physical and mental exercise methods in Chinese culture [32]. Both emphasize comprehensive training of the body (posture and movement), breathing (breath), and consciousness (mind) [33]. Previous studies have shown that TC and qigong have similar therapeutic potential in clinical research, so these two have become widely accepted methods in the research of physical and mental exercises [34, 35]. Secondly, yoga, and Pilates share similar training elements in modern clinical practice, including posture control, core muscle strengthening, breathing and body coordination, as well as body and mind awareness [36, 37]. Both are recognized as typical physical and mental exercise methods in modern rehabilitation practice [38, 39]. It is important to note that this classification is operational rather than historical. It does not involve any value judgments on the cultural or historical attributes of these patterns but is a descriptive classification based on the intervention characteristics of the studies included in this meta‐analysis.

## 3. Results

### 3.1. Literature Search

A total of 440 records were identified through searches across the five databases. Following the removal of 185 duplicates, 255 studies remained for title and abstract screening. Of these, 90 records were excluded as they were review articles or conference abstracts. The remaining 165 studies underwent full‐text review, and 145 were subsequently excluded based on the predefined eligibility criteria. Consequently, 20 studies were included for the final analysis. The complete study selection process is illustrated in Figure 1.

**FIGURE 1:**
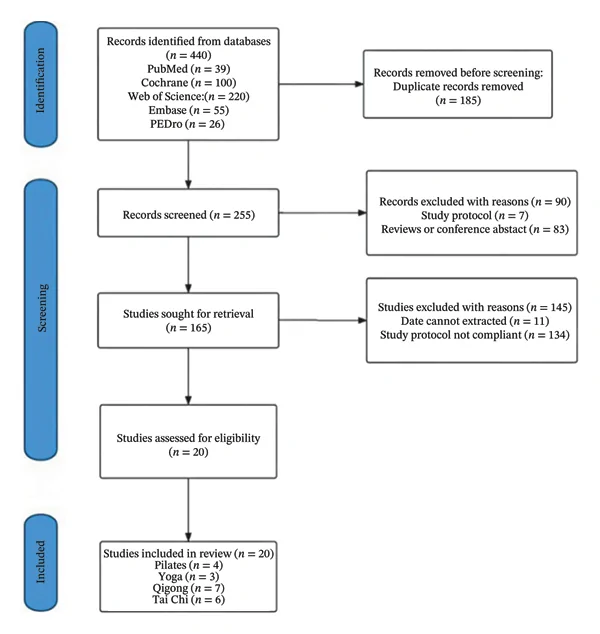
Referred reporting items for systematic reviews and meta‐analyses flowchart of study selection.

### 3.2. Characteristics of the Included Studies

As presented in Table 1, a total of 20 studies encompassing 1177 FM patients were identified. The United States accounted for the largest proportion of studies (*n* = 8) [13, 25, 40–45]. The remaining 12 studies originated from nine other countries: Turkey (*n* = 4) [14, 27, 46, 47] and one study each from Brazil [28], Germany [48], the United Kingdom [29], Canada [49], Italy [50], Iran [51], Spain [52], and Sweden [20].

Four MBE modalities were included in the analysis. The distribution of studies was as follows: TC was represented by 6 studies [13, 25, 29, 44, 45, 50], qigong by 7 studies [20, 41–43, 49, 51, 52], Pilates by 4 studies [14, 27, 28, 46], and yoga by 3 studies [40, 47, 48]. The intervention duration across all studies ranged from 4 to 32 weeks.

This analysis examined outcomes across seven domains: pain, overall impact of FM, depression, anxiety, fatigue, sleep, and quality of life. The assessment tools used in the included studies are summarized as follows:

Pain was evaluated in 19 studies using a range of instruments: the Visual Analog Scale (VAS) [14, 25, 27, 28, 41, 44, 45], Fibromyalgia Impact Questionnaire–pain subscale (FIQ–pain) [29], Revised Fibromyalgia Impact Questionnaire–pain subscale (FIQR–pain) [40], Short‐Form McGill Pain Questionnaire (SMPQ) [42], Visual Numerological Scale (VNS) [20], Short Form–36 pain subscale (SF–36 pain) [43], Widespread Pain Index (WPI) [50], Present Pain Intensity Scale (PPS) [48], Numeric Rating Scale for Pain Intensity (NRS–PI) [46, 47, 49, 52], and Brief Pain Inventory (BPI) [13].

The overall impact of FM was assessed in 17 studies using the FIQ or FIQR [13, 14, 25, 27–29, 40, 42–44, 46, 47, 49–52]. Depression was measured in 11 studies with instruments including the Hospital Anxiety and Depression Scale (HADS) [25, 41, 46, 50], Center for Epidemiologic Studies Depression Scale (CES‐D) [44, 48], depression subscales of FIQ or FIQR [29, 40], and Depression Anxiety Stress Scales–21 (DASS–21) [52] and Beck Depression Inventory (BDI) [20, 43]. Anxiety was evaluated in 10 studies using the State‐Trait Anxiety Inventory (STAI) [20, 27, 48], DASS–21 [52], HADS [25, 41, 46, 50], and anxiety subscales of the FIQ or FIQR [29, 40].

Sleep quality was examined in 10 studies, all employing the Pittsburgh Sleep Quality Index (PSQI) [13, 25, 28, 41, 42, 44, 48–51]. Fatigue was assessed in 5 studies via the fatigue subscales of the FIQ or FIQR [29, 40], Multidimensional Fatigue Inventory (MFI–20) [42], VAS fatigue [45], and Functional Assessment of Chronic Illness–Fatigue (FACIT–F) [50].

Finally, quality of life was evaluated in 9 studies using the Short Form–36 (SF–36) [25, 28, 44, 49, 50], Profile of Life Quality in Chronic Patients (PLC) [48], World Health Organization Quality of Life–BREF (WHOQOL–BREF) [20], Satisfaction with Life Scale (SWLS) [52], and the Quality‐of‐Life Scale (QOLS) [41].

### 3.3. Risk of Bias

Two researchers (ZPA and GJ) used the Cochrane risk of bias tool to assess the quality of the studies: random sequence generation, allocation concealment, blinding (participants, therapists, and assessors), incomplete outcome data, selective reporting, and other biases. The quality of each item was judged and classified as low risk of bias, uncertain risk of bias, and high risk of bias. The quality of the 20 studies included in this meta‐analysis is shown in Figure 2A,B.

**FIGURE 2:**
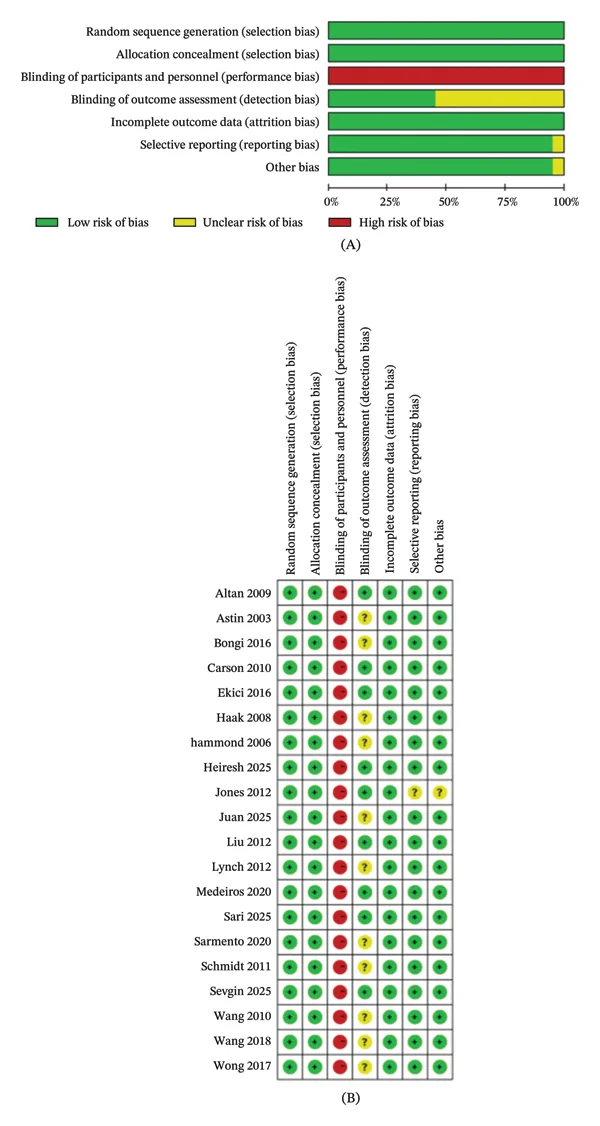
(A) Risk of bias graph and (B) risk of bias summary.

### 3.4. GRADE Evidence Quality Assessment

We assessed the certainty of evidence for each of the seven major outcomes (pain, overall FM impact, depression, anxiety, sleep, fatigue, and quality of life) using the Grading of Recommendations, Assessment, Development, and Evaluation (GRADE) approach. The assessment considered five domains: risk of bias (based on the Cochrane RoB results), inconsistency (based on *I* ^2^ statistics and subgroup analyses), indirectness, imprecision (based on sample sizes and 95% CIs), and publication bias.

The certainty of evidence ranged from moderate to very low. Pain, depression, and quality of life were rated as moderate certainty. Pain was downgraded due to substantial unexplained heterogeneity (*I* ^2^ = 83%), despite consistent direction of effects across studies. Depression was downgraded due to risk of bias in some studies, with no evidence of serious inconsistency (*I* ^2^ = 49%). Quality of life was downgraded for risk of bias, with heterogeneity (*I* ^2^ = 50%) being explained by sensitivity analysis.

FM impact, anxiety, and sleep were rated as low certainty, downgraded for both risk of bias and inconsistency. Fatigue was rated as very low certainty, downgraded for risk of bias, inconsistency, imprecision (limited sample size), and potential publication bias (fewer than 10 studies available). No indirectness was identified for any outcome, as all studies directly addressed the population, intervention, comparator, and outcomes of interest. The detailed GRADE assessment is presented in Supporting Table S4.

### 3.5. Primary Outcomes

Pain was assessed in 19 studies. Compared to control groups, MBE demonstrated significant improvements in pain reduction among FM patients (SMD = −0.72; 95% CI: −1.03 to −0.41; *p* < 0.01), albeit with considerable heterogeneity (*I* ^2^ = 83%; Figure 3). Sensitivity analyses conducted by sequentially removing individual studies did not substantially reduce the heterogeneity, and the overall result remained unchanged.

**FIGURE 3:**
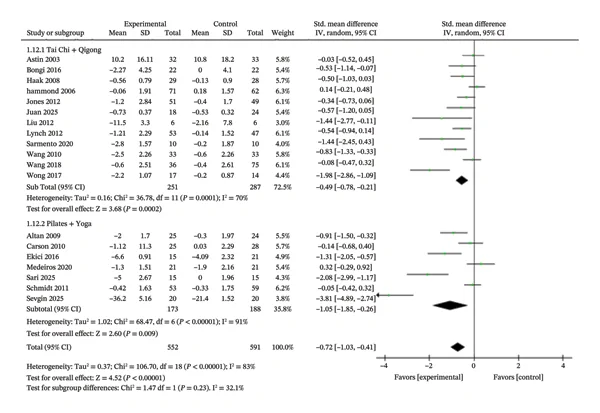
Effect of MBE in pain.

Subgroup analysis was performed to compare the effects of TC/qigong and Pilates/yoga on pain relief. Both subgroups demonstrated significant benefits: TC/qigong (SMD = −0.53, 95% CI: −0.81, −0.25; *p* < 0.01; *I* ^2^ = 70%; Figure 3) and Pilates/yoga (SMD = −1.05 (95% CI: −1.85, −0.26; *p* < 0.01; *I* ^2^ = 91%; Figure 3). However, there was no statistically significant difference in pain relief between the TC/qigong and Pilates/yoga subgroups (*p* = 0.23).

### 3.6. Secondary Outcomes

Seventeen studies assessed the functional status and overall impact of FM. Compared to control groups, MBE demonstrated a significant reduction in the overall impact of FM (SMD = −0.64; 95% CI: −0.93, −0.35; *p* < 0.01, Figure 4), with considerable heterogeneity observed (*I* ^2^ = 78%). Sensitivity analysis by sequential removal of individual studies did not substantially reduce the heterogeneity, and the overall finding remained robust. Subgroup analysis comparing TC/qigong and Pilates/yoga revealed that both significantly reduced the impact of FM. The pooled ES for TC/qigong (SMD = −0.51, 95% CI: −0.71, −0.31; *p* < 0.01; *I* ^2^ = 38%, Figure 4) and for Pilates/yoga (SMD = −1.16 (95% CI: −1.79, −0.53; *p* < 0.01; *I* ^2^ = 80%, Figure 4). There was no significant difference in the effect of FM remission between the TC/qigong subgroup and the Pilates/yoga subgroup (*p* = 0.05).

**FIGURE 4:**
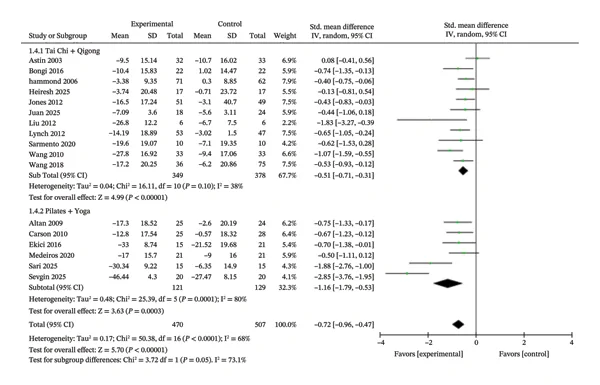
Effect of MBE in FM impact.

Only 10 studies assessed anxiety in patients with FM. Compared to control groups, MBE significantly alleviated anxiety symptoms (SMD = −0.58; 95% CI: −0.92, −0.24; *p* < 0.01, *I* ^2^ = 75%, Figure 5). Subgroup analysis revealed that both TC/qigong and Pilates/yoga were associated with significant improvements in anxiety symptoms (SMD = −0.58; 95% CI: −0.92, −0.24; *p* < 0.05; *I* ^2^ = 75%; Figure 5). The pooled ES for TC/qigong (SMD = −0.58, 95% CI: −1.13, −0.08; *p* < 0.05; *I* ^2^ = 83%), while for Pilates/yoga (SMD = −0.54,95% CI: −0.98, −0.10; *p* < 0.05; *I* ^2^ = 57%, Figure 5). No statistically significant difference was observed between the TC/qigong and Pilates/yoga subgroups regarding their effects on anxiety symptoms.

**FIGURE 5:**
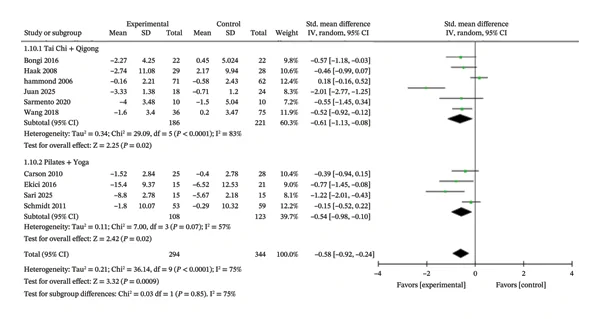
Effect of MBE in anxiety.

Eleven studies evaluated depressive symptoms in patients with FM. Compared with control groups, MBE was significantly more effective in alleviating depression (SMD = −0.41; 95% CI: −0.63, −0.20; *p* < 0.01, *I* ^2^ = 49%, Figure 6). Subgroup analysis revealed that Pilates/yoga did not show a significant improvement in depressive symptoms (SMD = −0.20; 95% CI: −0.48, 0.09; *p* = 0.17; *I* ^2^ = 0%, Figure 6), whereas TC/qigong demonstrated a significant beneficial effect (SMD = −0.49; 95% CI: −0.78, −0.21; *p* < 0.01; *I* ^2^ = 58%, Figure 6). Notably, no statistically significant difference was observed between TC/qigong and Pilates/yoga subgroups (*p* = 0.14).

**FIGURE 6:**
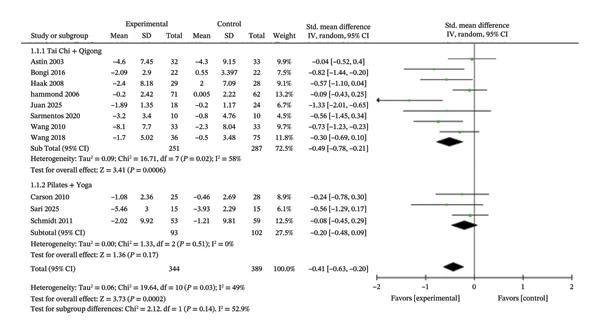
Effect of MBE in depression.

Ten studies evaluated sleep quality in patients with FM. The results indicated that MBE demonstrated a significant improvement in sleep quality compared to the control group (SMD = −0.36; 95% CI: −0.64, −0.08; *p* = 0.01, *I* ^2^ = 64%, Figure 7). A sensitivity analysis was conducted by excluding the study by Medeiros et al. [28]. This did not alter the direction or significance of the overall result but substantially reduced the heterogeneity (SMD = −0.45; 95% CI: −0.69, −0.22; *p* < 0.001; *I* ^2^ = 45%; Supporting Information 1).

**FIGURE 7:**
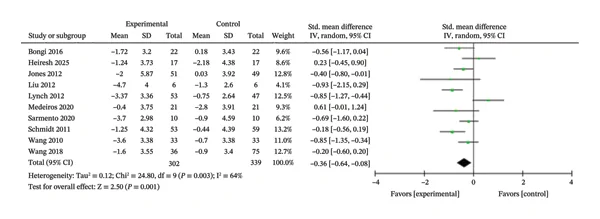
Effect of MBE in sleep.

Five studies evaluated the effect of MBE on fatigue in patients with FM. The results indicated that MBE was significantly more effective than the control group in alleviating fatigue (SMD = −0.98; 95% CI: −1.59, −0.38; *p* = 0.001, *I* ^2^ = 77%, Figure 8). Sensitivity analysis conducted by excluding the study by Wong et al. [45]. Revealed that the overall result remained largely unchanged; however, the heterogeneity was substantially reduced (SMD = −0.59; 95% CI: −0.90, −0.27; *p* < 0.001; *I* ^2^ = 20%; Supporting Information 2).

**FIGURE 8:**
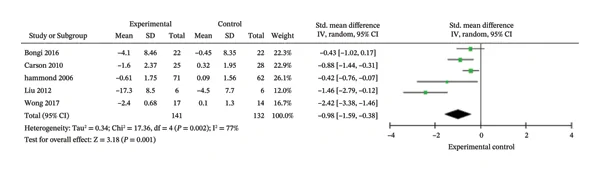
Effect of MBE in fatigue.

Nine studies evaluated the effect of MBE on quality of life in patients with FM. The pooled analysis indicated that MBE was associated with a significantly greater improvement in quality of life compared to the control group (SMD = 0.46; 95% CI: 0.22, 0.71; *p* < 0.01, *I* ^2^ = 50%, Figure 9). Sensitivity analysis demonstrated the exclusion of the study by Sarmento et al. [41]. Did not alter the significance of the overall effect; however, it led to a marked reduction in heterogeneity (SMD = 0.52; 95% CI: 0.31, 0.73; *p* < 0.001; *I* ^2^ = 32%; Supporting Information 3).

**FIGURE 9:**
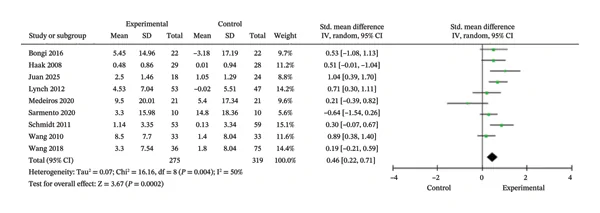
Effect of MBE in quality of life.

## 4. Discussion

This systematic review and meta‐analysis evaluated the comparative effectiveness of four prevalent MBE modalities—TC, qigong, Pilates, and yoga—for patients with FM. Six patient‐centered outcomes were assessed: overall FM impact (FIQ/FIQR), pain, fatigue, sleep disturbance, anxiety, and depression. Pooled results demonstrated that MBE interventions yielded significant improvements across all measured domains compared to control conditions. Subgroup analyses demonstrated that both TC/qigong and Pilates/yoga were effective in reducing pain, overall FM impact, and anxiety symptoms. Notably, while TC/qigong showed a statistically significant improvement in depressive symptoms, Pilates/yoga did not; however, the between‐subgroup difference was not statistically significant, indicating that the current evidence does not support the superiority of either modality in alleviating depression.

Apart from the overall therapeutic effect, it is important to analyze our research results in the context of existing evidence. Compared with the recent meta‐analysis published by Zhang et al. [53], our study differs in its analytical framework. Zhang et al. [53] conducted subgroup analyses by individual exercise type (TC, yoga, qigong, and Pilates separately), whereas we categorized the four modalities into two groups—TC/qigong and Pilates/yoga—for a head‐to‐head comparison based on their intrinsic attributes (theoretical foundations and training elements). This approach helps address the clinical question of whether MBEs with different theoretical underpinnings yield systematically different efficacy in FM patients. Furthermore, Zhang et al. used MD as the ES, which restricts inclusion to studies using identical assessment tools; in contrast, we used SMD, allowing us to pool data from studies employing diverse instruments and thus broaden the evidence base. In terms of evidence volume, our analysis included 20 studies with 1177 patients, yielding a larger average sample size per study compared to the 21 studies with 1155 patients in Zhang et al. Our study also systematically evaluated two previously under‐researched outcomes—anxiety and fatigue.

### 4.1. Effect of MBE on Pain

This study demonstrates that MBE significantly alleviates pain symptoms compared to control conditions. Specifically, both TC/qigong and Pilates/yoga modalities were effective in reducing pain among patients with FM, suggesting that MBE exhibits moderate‐to‐large potential for pain management in this population.

Among the four MBE modalities examined, TC and qigong are more widely applied in FM management, demonstrating significant improvements across all assessed outcomes. This efficacy may be attributed to their underlying mechanisms. FM is primarily conceptualized as a complex central pain disorder affecting pain perception, characterized by lowered pain thresholds and modulated by emotional, cognitive, and environmental factors [54]. Both TC and qigong, as traditional Chinese mind–body practices, integrate physical, psychosocial, emotional, spiritual, and behavioral components into a multidimensional intervention designed to influence both mind and body [55, 56], thereby yielding beneficial health outcomes [57, 58]. This integrative approach may elucidate the mechanisms underlying their analgesic effects. Research has indicated that combining TC with resistance training produces greater increases in pain threshold compared to resistance training alone, with significant main effects observed for time and group, as well as a significant time‐by‐group interaction, suggesting that TC substantially influences pain sensitivity reduction [59]. Additionally, studies on qigong have demonstrated its particular value in managing various chronic pain conditions [60–62], reducing analgesic medication dosage [62], potentially modifying inflammatory biology favorably [63], and conferring overall health benefits superior to medication alone [55].

Although yoga has been practiced for millennia [64], empirical evidence demonstrating its effects on individuals with persistent pain has only emerged in recent decades [65]. Contemporary yoga incorporates scientific elements such as Western gymnastics and anatomy, distinguishing it from its ancient origins and giving rise to diverse schools and practice styles [66]. Accordingly, this study categorized yoga alongside Pilates as grouped yoga and Pilates together as the Pilates/yoga category. Yoga and Pilates share characteristics similar to TC and qigong, emphasizing physical movement, breathing exercises, relaxation, meditation, and lifestyle modifications [15, 28, 67]. Both modalities have been shown to reduce muscular tension and somatic complaints [68, 69], increase acceptance rates of pain coping strategies [70], modify body awareness, and enhance pain acceptance [71, 72]. In line with recent reviews on mindfulness‐based interventions [53, 73], this meta‐analysis corroborates the beneficial effects of MBE for FM patients. Importantly, by performing subgroup analyses to evaluate the comparative efficacy of TC/qigong versus Pilates/yoga, our study provides nuanced insights that may guide more targeted clinical recommendations.

### 4.2. Effect of MBE on FM Impact, Fatigue, Sleep, Depression, Anxiety, and Quality of Life

The results of this study show that MBE can significantly improve the function, overall impact, and ADL of patients with FM compared with the control group. Some studies have shown that FIQ or FIQR scores are positively correlated with the quality of life. MBE can improve the quality of life of FM patients by reducing the symptoms of FM (such as fatigue, sleep, depression, and anxiety) [74, 75]. However, Sarmento et al. [41] found that qigong could improve the symptoms of FM, but in terms of ADL, the improvement of the control group was more obvious than that of the qigong group. The difference between FM symptom improvement and quality of life results may be related to the small sample size and the tools used to evaluate quality of life in the study.

In terms of fatigue, FM patients often have a high level of fatigue [76]. FM fatigue may be peripheral fatigue or central fatigue. The first fatigue is due to neuromuscular dysfunction, while the second fatigue means changes in neuroendocrine and neurotransmitter systems, which makes FMS a very disabling state [77]. A previous study using magnetic resonance spectroscopy (MRS) to measure the recovery rate of phosphocreatine (CP), a reliable indicator of skeletal muscle mitochondrial metabolism, in the thigh muscle of subjects found that CP recovery was significantly increased and the recovery time of CP was decreased after 12 weeks of TC training, indicating that TC training accelerated mitochondrial ATP synthesis and improved muscle mitochondrial function in the elderly [78]. This may be one of the mechanisms by which MBE improves fatigue. Our results show that FM has significant positive changes in fatigue level after MBE intervention.

In terms of sleep quality, some studies have speculated that the sleep quality of FM patients is related to other symptoms, such as pain, anxiety, and depression [50]. In many MBE studies, it was found that the improvement of pain, depression, and anxiety in patients with FM was positively correlated with sleep quality [25, 48, 50, 75, 79]. Studies have also found that MBE activates parasympathetic nerves and improves sleep through periodic breathing and rhythmic muscle contractions [80, 81]. Therefore, the results of this study show that MBE can improve the sleep quality of FM patients. This result is not surprising.

Among the patients with FM, the prevalence of emotional disorders such as depression and anxiety is higher than that of the general population and other rheumatic patients [82, 83]. The patients with FM may have symptoms such as increased functional disability, decreased energy, decreased leisure interest, decreased motivation, and problems in interpersonal relationships, which may reduce the quality of life and may cause psychological pain [28]. Some studies have shown that MBE can significantly improve the depression and anxiety of FM patients after intervention [25, 40, 43]. In addition, MBE can regulate the attention control network and relieve the symptoms of depression and anxiety [84]. Some studies have shown that TC can significantly regulate the brain structure of areas related to emotional regulation, such as the insula and putamen [85, 86], and also regulate the functional connection of key areas of the cognitive (attention) control network, such as the DLPFC [87]. Additionally, subgroup analysis indicated that Pilates/yoga failed to achieve significant improvements in depression and anxiety, though no significant difference emerged between TC/qigong and Pilates/yoga. This null finding may stem from limited sample sizes and inconsistent assessment tools across studies. Therefore, additional investigations employing larger cohorts and uniform outcome measures are needed to validate the effects of Pilates/yoga on psychological symptoms in this population.

### 4.3. Sources of Heterogeneity

Substantial statistical heterogeneity was observed across several outcomes in this meta‐analysis (*I* ^2^ ranging from 64% to 83%), which represents an important limitation of our findings. We explored potential sources through sensitivity analyses by sequentially removing individual studies; however, the heterogeneity remained largely unchanged for most outcomes, suggesting that no single study was solely responsible for the observed variability.

The heterogeneity likely arises from multiple sources. First, intervention protocols varied considerably across the included studies, including differences in exercise frequency (ranging from once weekly to daily sessions), session duration (20–60 min per session), and total intervention length (4–32 weeks). Second, control group types were heterogeneous, with some studies using waitlist controls, others using health education, and still others using active exercise comparators (e.g., aerobic exercise or usual care). The choice of control condition may influence the magnitude of the between‐group ESs. Third, outcome assessment tools were not uniform across studies—for instance, pain was measured using VAS, NRS, BPI, SF–36 pain subscale, and other instruments, each with different scoring ranges and sensitivity. Although sleep quality was consistently assessed using the PSQI, the scoring dimensions and cutoff values varied. Fourth, patient populations differed across studies in terms of disease duration, baseline symptom severity, and concomitant medication use, all of which may affect treatment response.

Due to the limited number of studies available for each outcome (maximum 19 for pain, minimum 5 for fatigue), we were unable to perform meta‐regression or more detailed subgroup analyses to quantitatively explore these sources of heterogeneity. Future studies with larger sample sizes and more standardized intervention protocols and outcome measures are needed to better understand the factors contributing to heterogeneity in MBE research for FM.

### 4.4. Future Directions

The optimal duration of MBE interventions for FM patients warrants further investigation. Moreover, well‐designed multigroup RCTs are needed to elucidate the comparative effectiveness of different MBE modalities in treating FM.

### 4.5. Study Limitations

Several limitations of this study should be acknowledged. First, the rapid accumulation of new evidence represents a limitation. At the time this study was finalized, another meta‐analysis on the same topic was published [53]. Although there are clear differences in the analysis framework (systematic comparison vs. individual effect assessment), ES (SMD vs. MD), and outcome focus between the two, the overlapping subject areas require readers to make a comprehensive judgment when interpreting the conclusions of this study by integrating this period’s evidence for a more accurate assessment in order to avoid regarding the results of a single meta‐analysis as a definitive conclusion. Second, the absence of subgroup analysis based on intervention duration precludes the provision of reliable evidence regarding optimal treatment length for MBE in FM patients. Third, the current findings do not establish clear criteria for selecting among different MBE modalities, which poses a practical challenge for patients who must consider factors such as economic costs and personal preferences when choosing an appropriate intervention. Finally, the limited number of high‐quality RCTs investigating yoga and Pilates restricted our ability to perform comprehensive multidimensional subgroup analyses for Pilates/yoga modalities.

## 5. Conclusions

Findings from this meta‐analysis indicate that TC, qigong, Pilates, and yoga significantly alleviate core symptoms of FM, including pain, fatigue, sleep disturbance, anxiety, depression, and overall impact. Subgroup analyses demonstrated comparable efficacy between TC/qigong and Pilates/yoga in reducing pain, overall impact, and anxiety. While TC/qigong showed significant antidepressant effects, no significant difference was detected between subgroups, suggesting that current evidence does not establish the superiority of TC/qigong over Pilates/yoga for depression management in FM.

## Funding

No funding was received for this manuscript.

## Conflicts of Interest

The authors declare no conflicts of interest.

## Supporting Information

Additional supporting information can be found online in the Supporting Information section.

## Supporting information


**Supporting Information** Supporting Information 1: Results of sensitivity analysis for sleep quality; Supporting Information 2: Results of sensitivity analysis for fatigue; Supporting Information 3: Results of sensitivity analysis for quality of life. PubMed Search History—Supporting Table S1; Cochrane history—Supporting Table S2; Embase history—Supporting Table S3; GRADE—Supporting Table S4. PRISMA_2020_checklist.

## Data Availability

The data that support the findings of this study are available from the corresponding author upon reasonable request.
