# Supplementary material for: The Effects of Mind–Body Exercise on the Management of Fibromyalgia Symptoms: A Systematic Review and Meta‐Analysis
Source: Pain Res Manag. 2026 Nov 15;2026:9436329. doi: 10.1155/prm/9436329 (PMC13770289; doi:10.1155/prm/9436329)
Supplement: Supplementary file 1 [file PRM-2026-9436329-s001.zip › GRADE-Supplementary Table S4.docx]

| **Certainty assessment** | | | | | | | **№ of patients** | | **Effect** | | **Certainty** | **Importance** |
| --- | --- | --- | --- | --- | --- | --- | --- | --- | --- | --- | --- | --- |
| **№ of studies** | **Study design** | **Risk of bias** | **Inconsistency** | **Indirectness** | **Imprecision** | **Other considerations** | **[干预]** | **[对照]** | **Relative (95% CI)** | **Absolute (95% CI)** |  |  |
| **Pain** | | | | | | | | | | | | |
| 19 | randomised trials | not serious | serious | not serious | not serious | none | 552 | 591 | - | SMD **0.72 SD lower** (1.03 lower to 0.41 lower) | ⨁⨁⨁◯ Moderate | CRITICAL |
| **FM impact** | | | | | | | | | | | | |
| 17 | randomised trials | serious | serious | not serious | not serious | none | 470 | 507 | - | SMD **0.72 SD lower** (1.03 lower to 0.41 lower) | ⨁⨁◯◯ Low | CRITICAL |
| **Anxiety** | | | | | | | | | | | | |
| 10 | randomised trials | serious | serious | not serious | not serious | none | 294 | 344 | - | SMD **0.58 SD lower** (0.92 lower to 0.24 lower) | ⨁⨁◯◯ Low | CRITICAL |
| **Depression** | | | | | | | | | | | | |
| 11 | randomised trials | serious | not serious | not serious | not serious | none | 344 | 389 | - | SMD **0.41 SD lower** (0.63 lower to 0.2 lower) | ⨁⨁⨁◯ Moderate | CRITICAL |
| **Sleep** | | | | | | | | | | | | |
| 10 | randomised trials | serious | serious | not serious | not serious | none | 302 | 339 | - | SMD **0.36 SD lower** (0.64 lower to 0.08 lower) | ⨁⨁◯◯ Low | CRITICAL |
| **Fatigue** | | | | | | | | | | | | |
| 5 | randomised trials | serious | serious | not serious | serious | none | 141 | 132 | - | SMD **0.98 SD lower** (1.59 lower to 0.38 lower) | ⨁◯◯◯ Very low | CRITICAL |
| **Quality of life** | | | | | | | | | | | | |
| 9 | randomised trials | serious | not serious | not serious | not serious | none | 275 | 319 | - | SMD **0.46 SD higher** (0.22 higher to 0.71 higher) | ⨁⨁⨁◯ Moderate | CRITICAL |

**CI:** confidence interval; **SMD:** standardised mean difference;

¹ Downgraded by one level for serious inconsistency（*I²* > 50% not explained by subgroup/sensitivity analyses）.

² Downgraded by one level for serious risk of bias（methodological limitations in allocation concealment and/or blinding， particularly in early or small-sample studies）.

³ Downgraded by one level for serious imprecision（total sample size < 400， 95% CI wide）.

⁴ Downgraded by one level for serious publication bias（fewer than 10 studies， funnel plot testing not feasible）.
